# COVID-19: A new digital dawn?

**DOI:** 10.1177/2055207620920083

**Published:** 2020-04-11

**Authors:** Tim Robbins, Sarah Hudson, Pijush Ray, Sailesh Sankar, Kiran Patel, Harpal Randeva, Theodoros N Arvanitis

**Affiliations:** 1 University Hospitals Coventry & Warwickshire NHS Trust, UK; 2 Institute of Digital Healthcare, WMG, University of Warwick, UK; 3 Bristol Heart Institute, United Hospitals Bristol NHS Foundation Trust, UK; 4 Warwick Medical School, University of Warwick, UK

The Coronavirus disease (COVID-19) pandemic represents an unprecedented challenge for healthcare systems internationally. On 25 March 2020, the World Health Organisation reported 413,467 confirmed cases and 18,433 deaths.^1^ The disease has been identified as highly infective, causing a range of symptoms from asymptomatic infection to respiratory failure or death.^2^ The disease appears to be particularly severe in the elderly and those with underlying health conditions.^3^ Healthcare systems have had to adapt rapidly to the evolving situation for three main reasons: firstly, there is a need to triage and treat large numbers of patients with respiratory illness;^4^ secondly, there is a need to protect the healthcare workforce to ensure they are able to treat the sick;^5,6^ and thirdly we need to shield the elderly and most vulnerable from becoming infected.^7^

This triumvirate of aims has required rapid and wide reaching innovation, in order to implement successful strategies to hopefully, in time, overcome the COVID-19 pandemic. This drive for innovative working approaches has resulted in remarkable advances in the use of digital health. Such approaches have both developed organically or been implemented with centralised support. They fall into three broad categories: digital communication strategies, digital educational initiatives and digital patient management solutions.

## Communication

The rapidity with which COVID-19 has spread globally, alongside the novelty of the virus, has required innovative responses. The continual flow of new information and novel ways of practicing have resulted in the development of new digital communication strategies. Clinical groups report the widespread adoption of messaging tools, such as WhatsApp and Slack, for communication, in order to organise service provision or manage staff rotas, in the face of high levels of staff sickness or self-isolation.

Social media is becoming a particularly important part of professional communication across multiple platforms such as Facebook and Twitter. The largest of these groups in the United Kingdom (UK) is the ‘COVID Doctors Forum (UK)’ administered by the Doctor’s Association UK, which on 23 March had 11,354 members.^8^ Discussions on the platform have covered a range of topics essential to healthcare staff, including use/availability of personal protective equipment, procedures for self-isolation and lessons from colleagues internationally. Blog posts have also been particularly prevalent, including one by Health Education England Topol Digital Health Fellow Sarah Hudson, who outlines a cardiology approach to managing staff safety during the coronavirus pandemic.

Enhanced digital communication has also developed through more structured formats, including the Discourse Digital Health Network, which is a ‘Discussion and collaboration for UK and international digital health communities’.^9^ By 23 March, there were 27 threads considering the digital response to COVID-19, supported by active discussion from senior digital health leaders. Similarly, on 24 March, NHX participated in a live webinar on the digital response to the pandemic; a meeting that perhaps, previously, would have been designed as a face-to-face conference discussion.

## Education

Educational activities have been particularly hard hit during the Coronavirus pandemic, with the widespread cancellation of conferences, training courses and postgraduate examinations.^10^ There has, however, been the need for rapid education of the healthcare workforce in how best to manage the respiratory conditions encountered, as well as a need to provide redeployment education to staff who change roles during the pandemic management approach. This has resulted in the application of innovative digital health solutions to provide educational content and continuity. In the United Kingdom, for example, doctors in training will have their Annual Review of Clinical Progression (ARCP) assessments held virtually, alongside a range of other virtual approaches to protect trainees and their progression during the pandemic.^11^ Alongside this, a range of innovative e-learning packages have been produced far quicker than digital content would be usually, in order to facilitate upskilling of the healthcare workforce in the treatment of viral respiratory illnesses. There are good examples of innovative e-learning packages to support preparedness, including, as an example, a bespoke redeployment training package that was created in less than 72 h at University Hospitals Coventry and Warwickshire (UHCW) NHS Trust, in the West Midlands region, UK.^12^

## Patient management

Perhaps the greatest digital health transformation has been the rapid development and implementation of new models of care, supported by digital health innovation. These digital approaches have arisen out of a need to shield vulnerable patients from being exposed to the risks of coming into hospital, promoting social distances and protecting staff. In the UK, this has been facilitated across both primary and secondary care by the use of telemedicine consultation approaches.^13^ There has been significant innovation and support from the MedTech sector, with the rapid rollout of digital tools and packages. For example, EMIS (Egton Medical Information Systems), the UK’s largest supplier of electronic health records to primary care, has introduced a range of interventions, including modifying coding, alert tracking and the enabling of video consultations, free of charge to all EMIS web general practitioner (GP) practices in the UK.^14^ Similarly, NHS Trusts have been rushing to implement remote consultation solutions. The vast majority of clinic attendances have switched to remote consultation methods, ranging from basic telephone-based consultations to more complex video-conference based telemedicine or App based solutions.^15^ Multi-disciplinary team meetings have switched from in-person attendance to Zoom and other platforms to facilitate complex care decisions being made without risking physical gatherings of large groups of healthcare staff. The implementation of these digital care processes has required the rapid navigation of governance and digital integration, which has been facilitated by the urgent need created by the COVID-19 pandemic. Without this, implementation may have been significantly slower.

## Conclusion

The COVID-19 pandemic will have wide-ranging impacts across healthcare, the economy and society as a whole. The human costs of the disease will unfortunately be very high and long remembered. Despite this, through such adversity, the healthcare system that works to protect us may become stronger and more robust. A central foundation of this change will be the development and implementation of new ways of remote and digital health working. Adversity has long been an important driver of innovation and modernisation of healthcare, with previous such lessons typically learnt periods of conflict and warfare (such as casualty clearing stations and modern blood transfusion practice).^16,17^ We must ensure we learn from this period of adversity in the same way, and look to sustainably embed this new dawn of digital health practices in the care models of the future.
